# What is important to the decision to disclose nonsuicidal self‐injury in formal and social contexts?

**DOI:** 10.1002/jclp.23503

**Published:** 2023-03-06

**Authors:** Sylvanna Mirichlis, Mark Boyes, Penelope Hasking, Stephen P. Lewis

**Affiliations:** ^1^ Discipline of Psychology, School of Population Health Curtin University Perth Australia; ^2^ Curtin enAble Institute, Faculty of Health Sciences Curtin University Perth Australia; ^3^ Department of Psychology University of Guelph Guelph Canada

**Keywords:** nonsuicidal self‐injury disclosure, self‐injury disclosure, voluntary self‐disclosure

## Abstract

**Objective:**

Disclosure of nonsuicidal self‐injury (NSSI) is associated with a range of both positive (e.g., help‐seeking) and negative (e.g., discrimination) outcomes. The aim of this study was to assess the importance of a range of factors concerned with: NSSI experiences, self‐efficacy to disclose self‐injury, interpersonal factors, and reasons for or expectations of disclosure, to the decision to disclose self‐injury to friends, family members, significant others, and health professionals.

**Methods:**

Three hundred seventy‐one participants with lived experience of NSSI completed a survey in which they rated the importance of the aforementioned factors to the decision of whether to disclose NSSI to different people. A mixed‐model analysis of variance was conducted to investigate whether the factors differed in importance and if this importance differed across relationship types.

**Results:**

All factors held importance, though to differing degrees, with those related to relationship quality being most important overall. Generally, factors relating to tangible aid were considered more important when considering disclosure to health professionals than to other people. Conversely, interpersonal factors, particularly trust, were more important when disclosing to individuals in social or personal relationships.

**Conclusion:**

The findings provide preliminary insight into how different considerations may be prioritized when navigating NSSI disclosure, in a way that may be tailored to different contexts. For clinicians, the findings highlight that clients may expect tangible forms of support and nonjudgment in the event that they disclose their self‐injury in this formal setting.

## 1 INTRODUCTION

Nonsuicidal self‐injury (NSSI) is the intentional damage caused to a person's own body that is not suicidal in nature, nor does it align with their particular cultural or societal norms (e.g., cutting, burning, and self‐battery; International Society for the Study of Self‐Injury, 2022; Swannell et al., 2014). There are many functions that NSSI may serve, including both intrapersonal functions, such as to regulate one's emotions, and interpersonal functions, for example seeking support (Taylor et al., 2018). Further, the behavior is associated with a number of challenging experiences including mental health difficulties and later suicidal ideation and behavior (Kiekens et al., 2021; Klonsky et al., 2014). Given that approximately 5% of adults, 13% of young adults, and 17% of adolescents have lived experience of NSSI, many people are likely to know or come to know someone who has self‐injured (Swannell et al., 2014).

NSSI disclosure can be associated with a number of potential positive outcomes including social and professional support, and self‐advocacy (Burke et al., 2019; Rosenrot & Lewis, 2020). In these ways, disclosing one's experience of NSSI could contribute to opportunities to mitigate negative outcomes associated with self‐injury whether that be in accessing interventions and/or addressing stigma. However, disclosure of self‐injury is a complex phenomenon with various barriers such as stigma, and anticipated impact on the recipient of the disclosure, being identified (Simone & Hamza, 2020).

Among people commonly disclosed to are friends, significant others, and family members, and in formal settings, health professionals such as psychologists (Simone & Hamza, 2020). Disclosures of NSSI are associated with characteristics of the behavior, including the function of NSSI and visibility of scars, (Mirichlis et al., 2022; Simone & Hamza, 2021). The nature of a disclosure experience may differ depending on the setting; for example, disclosing one's self‐injury online can provide anonymity that is difficult to achieve face‐to‐face (Frost et al., 2016). While disclosure of self‐injury may be a first step in seeking support or be motivated by the need for medical care (Armiento et al., 2014; Hasking et al., 2015), NSSI stigma and internalized shame have been identified as barriers to disclosing one's self‐injury (Long, 2018; Rosenrot & Lewis, 2020). Similarly, the anticipated impact on the recipient of the disclosure (e.g., distress, placing burden), and the individual's relationship with them, may factor into whether, and to whom, someone discloses a history of self‐injury (Armiento et al., 2014; Mirichlis et al., 2022; Simone & Hamza, 2020).

While existing literature has provided valuable insight into factors associated with disclosing NSSI, relatively little is known about what considerations inform the *decision* to voluntarily disclose one's experience of self‐injury, or how these factors may be prioritized in the decision to disclose (Simone & Hamza, 2020). The Disclosure Decision‐Making (Greene, 2009) and Disclosure Processes models (Chaudoir & Fisher, 2010) outline key factors that could be important in this regard. In these models, it is proposed that before deciding whether to share sensitive personal information individuals evaluate: aspects of the information itself (including potential stigma, course of the behavior, visibility); who to tell and why (e.g., the information is relevant to them, nature of the relationship); their own self‐efficacy to disclose the information; and potential outcomes or goals of the disclosure (Chaudoir & Fisher, 2010; Greene, 2009). This cognitive decision‐making process is seen as integral in the disclosure of personal information, and may also underlie the decision to disclose a history of NSSI (Mirichlis et al., 2022). However, this approach and the importance of the factors within these models to the decision to disclose self‐injury are yet to be investigated (Mirichlis et al., 2022).

Beyond those outlined in the above models, several other factors may be relevant to decision‐making concerning NSSI disclosure, including NSSI‐related factors (e.g., visibility of scars), interpersonal factors (e.g., trust), reasons for disclosure (e.g., seeking help), and/or expectations of disclosure (e.g., experiencing stigma; Simone & Hamza, 2020). Understanding what individuals consider to be important to the decision of whether to disclose NSSI could aid in supporting individuals navigating disclosures, across social (e.g., to friends and loved ones) and more formal contexts (e.g., clinical settings and workplaces).

Following the above, we examined the relative importance of factors such as stigma, NSSI experiences, disclosure self‐efficacy, relationships, expectations, and goals of disclosure in the decision of whether to disclose NSSI to different people. We hypothesized that stigma‐related factors, the perceived visibility of one's NSSI, and factors related to support‐seeking would be rated among the most important factors to the decision (Simone & Hamza, 2020). We also predicted that the importance of factors would vary depending on the prospective disclosure recipient; for example, we expected that concerns about how the disclosure might impact the recipient or the individual's relationship would be less important when disclosing to a professional, compared to when disclosing to a friend.

## 2 MATERIALS AND METHODS

### 2.1 Participants

The sample comprised 371 individuals in Australia with lived experience of NSSI, 89 of whom were university students who received credit points for participation. The majority of the sample identified women (80.6%), 7.5% identified as men, and 11.9% identified as another gender, commonly nonbinary. The age of participants ranged from 17 to 72 years (*M* = 23.94, SD = 6.33). The majority (87.33%) of the sample were born in Australia, with 4.0% identifying as Aboriginal or Torres Strait Islander. The majority of the sample (79.8%) indicated having at least one mental illness diagnosis, with the three most commonly reported diagnoses being related to depression (*n* = 202), anxiety (*n* = 196), and posttraumatic stress (*n* = 80).

### 2.2 Measures

#### 2.2.1 Demographics and mental illness

In the first block of questions, participants were asked about their age, gender identity, country of birth, and whether they identified as Aboriginal or Torres Strait Islander. Participants were also asked to indicate whether they had any mental illness diagnoses and if so what the diagnosis was, as well as whether they had ever sought professional help (and from whom) for their mental health.

#### 2.2.2 NSSI

After confirming that they had a history of self‐injury, participants were asked the number of times that they had self‐injured within the last year. The Inventory of Statements About Self‐Injury (ISAS; Klonsky & Glenn, 2009) was used to assess which methods of NSSI participants had engaged in and which (if any) of these were their primary form of self‐injury; the ages at which they first and most recently self‐injured; whether they experience physical pain when they self‐injure, and whether they are alone when they self‐injure. Good test–retest reliability has been established for this part of the ISAS (*r* = 0.85; Klonsky & Olino, 2008). Participants were asked whether they had ever sought professional help for self‐injury before being presented the second section of the ISAS, which assesses NSSI functions on a scale from 0: not relevant to 2: very relevant. Good test–retest reliability and internal consistency has been demonstrated for the function subscales (*r* = 0.60–0.82, *α* = 0.80–0.87; Glenn & Klonsky, 2011; Klonsky & Glenn, 2009); in the current sample Cronbach's *α* for intrapersonal functions was *α* = 0.63 and for interpersonal functions *α* = 0.76.

#### 2.2.3 Disclosure experience and decision‐making

Participants were asked whether they had ever voluntarily disclosed their self‐injury to another person face‐to‐face, and if so to whom (i.e., friend, family member, significant other, health professional, or other). All participants were then presented with a range of factors (https://doi.org/10.23668/psycharchives.12212) informed by previous NSSI disclosure literature (e.g., Simone & Hamza, 2020) and broader models of disclosure of personal information (Chaudoir & Fisher, 2010; Greene, 2009). These factors were conceptualized as “Considerations about NSSI” including stigma, course, and visibility; “Self‐Efficacy” to disclose; “Interpersonal” considerations (e.g., relationship quality); and “Reasons/Expectations of Disclosure,” such as seeking professional help, or expecting a particular reaction (Chaudoir & Fisher, 2010; Greene, 2009; Simone & Hamza, 2020). Each factor was rated on a scale from 0 = not at all important to 100 = extremely important to the decision to disclose NSSI, and rated separately for disclosure to friend, family member, significant other, and health professional. The use of these relationship types was informed by existing NSSI disclosure literature (Simone & Hamza, 2020). If participants had never disclosed their NSSI, they were asked to imagine how important each factor would be to that decision.

### 2.3 Procedure

Upon gaining ethical approval, the study was advertised via a university student research participation pool and social media pages. Participants recruited via the university pool were granted course credit. The advertisements linked individuals to the online survey on Qualtrics; here, they were presented further information about the study and provided informed consent to participate. The survey took approximately 30 min to complete. At the end of the study, participants were debriefed and provided resources and contacts for support services.

### 2.4 Analyses

A mixed‐model analysis of variance (ANOVA) was used to investigate whether the decision factors differed in importance, that is, whether this importance differed across relationship type, in general, and whether the importance of each factor differed across relationship types. Post‐hoc ANOVAs were used to investigate the nature of significant interactions. Given the number of comparisons, a conservative *α* level of 0.01 was used for these post‐hoc analyses (Streiner, 2015).

## 3 RESULTS

### 3.1 NSSI characteristics

The average age at NSSI onset was 13.83 years (SD = 3.56), with the highest reported age of onset being 40 years. The majority of participants (71.43%; *n* = 265) had self‐injured at least once in the past year, with 150 of these individuals reportedly self‐injuring at least five times in this timeframe. The three most commonly reported methods of self‐injury were; cutting (*n* = 311), self‐battering (*n* = 220), and severe scratching (*n* = 212). The most strongly endorsed function of NSSI was affect regulation (*M* = 4.97, SD = 1.20).

### 3.2 Recipients of disclosure

The majority of the sample (81.2%) reported that they had previously disclosed their self‐injury to at least one person and 42.0% indicated having previously sought professional help for NSSI specifically (compared to 88.4% who sought professional help for mental health difficulties generally). Of these disclosures, 63.6% were to friends, 54.7% were to health professionals, 47.7% were to one's significant other, 34.0% were to family members, and a further 5.1% were to other recipients such as strangers, people in their workplace, and teachers.^1^ Psychologists/therapists (*n* = 87), psychiatrists (*n* = 48), and medical doctors including specialists (*n* = 72) were among the most commonly disclosed to health professionals, though disclosures to counselors (*n* = 24), nurses (*n* = 10), and other allied‐health workers (*n* = 2) were also reported.

## 4 MAIN EFFECTS OF VARIANCE OF IMPORTANCE

Given within‐group variance was not equal for each group of disclosure recipients, the Huynh–Feldt epsilon was used when interpreting main effects. There was a main effect of the importance of the various factors to the decision to disclose NSSI regardless of whom the disclosure would be to, *F* (25.96, 38,415.14) = 118.324, *p* < 0.001, partial *η* ^2^ = 0.074. On average, the factor rated most important was relationship trust (*M* = 78.59, SD = 30.04); rated least important was expecting the relationship with the person to be positively impacted by the disclosure (*M* = 40.70, SD = 34.17). There was a between‐groups effect such that, on average, the factors considered important differed across recipients of disclosure regardless of decision factor (*F* (3, 1480) = 22.89, *p* < 0.001, partial *η* ^2^ = 0.044). Yet, there was a significant interaction effect, indicating that the importance of individual factors to the decision to disclose NSSI differed depending on whom the disclosure would be to, *F* (77.87, 38,415.14) = 29.119, *p* < 0.001, partial *η* ^2^ = 0.056.

### 4.1 Post‐hoc comparisons of importance across disclosure recipients

The interaction effects (*α* = 0.01) show the way that the importance of the factors differ across relationship types. These effects highlight the variability in the importance of factors across relationship types (https://doi.org/10.23668/psycharchives.12212). All factors significantly differed by relationship type, apart from being seen engaging in NSSI (*F* (3, 1480) = 2.01, *p* = 0.110, partial *η* ^2^ = .004), being confident in one's own knowledge to answer questions about NSSI (*F* (3, 1480) = 0.106, *p* = 0.957, partial *η* ^2^ = 0.000), having previously disclosed mental health difficulties (*F* (3, 1480) = 1.741, *p* = 0.157, partial *η* ^2^ = 0.004), confidence in being able to disclose NSSI (*F* (3, 1480) = 3.788, *p* = 0.010, partial *η* ^2^ = 0.008, having wounds seen (*F* (3, 1480) = 2.694, *p* = 0.045, partial *η* ^2^ = 0.005), and feeling they had recovered *F* (3, 1480) = 3.162, *p* = 0.024, partial *η* ^2^ = 0.006.

Of the 38 factors that differed by relationship type, 36 featured a difference between a health professional and other relationship types. For example, while the amount of trust in the relationship was rated most important overall, when compared to the other relationship types the rating for this factor for health professionals was the lowest (*M* = 71.17, SD = 31.88). A similar trend can be observed for other interpersonal factors. In contrast, seeking tangible aid as a reason for disclosure was more important when disclosing to health professionals. For example, “wanting to seek professional help” was rated higher when disclosing to health professionals (*M* = 77.63, SD = 30.04) compared to disclosing to the other relationship types (overall *M* = 59.46, SD = 35.07). Though there were differences in importance of the factors among the social relationships (friends, significant other, and family), the only factor to differ between all four relationship types was: “Knowing whether this person has also self‐injured.” This factor was most important when considering disclosing to a friend.

While friends and significant others were the most similar of the groups, the mean importance did differ on six factors. Seeking professional help, the intensity of the NSSI, relevance of their NSSI to the disclosure recipient, the prospect of telling the recipient before they otherwise found out about their self‐injury, and the impact of not telling this person were rated as being more important to the decision to disclose to a significant other compared to a friend. In contrast, knowing that the disclosure recipient had also self‐injured was considered to be more important when disclosing to a friend, than to a significant other. The most important factor when considering disclosing to a friend was the quality of the relationship (*M* = 82.94, SD = 24.98), for family it was wanting to conceal their NSSI (*M* = 78.47, SD = 30.41), and for significant others it was the amount of trust in the relationship (*M* = 83.19, SD = 27.99).

## 5 DISCUSSION

The aim of this study was to assess the relative importance of factors considered in the decision of whether to voluntarily disclose a history of NSSI to friends, family members, significant others, and health professionals. The factors investigated were drawn from NSSI disclosure literature as well as theoretical accounts of disclosing personal information (Chaudoir & Fisher, 2010; Greene, 2009; Simone & Hamza, 2020). Gaining a better understanding of how such considerations may be prioritized when disclosing to different people could hold implications for how disclosures may be better responded to in both social and formal contexts.

Although all factors were rated as important (with several factors not significantly differing across disclosure recipients) for the most part the extent of importance did vary. This suggests individuals make different cognitive evaluations as part of navigating whether they would disclose NSSI with a particular person, consistent with models of disclosure of personal information and recent NSSI disclosure research (Chaudoir & Fisher, 2010; Greene, 2009; Mirichlis et al., 2022). For example, an individual may be more inclined to disclose to someone with whom they have a highly trusting relationship, regardless of whether other people already knew about their self‐injury. Given the complexity of NSSI disclosure, understanding what individuals consider to be important to the decision to disclose NSSI and eliciting what is relevant when disclosing to different people could be helpful in guiding individuals with lived experience of NSSI through the decision‐making process, potentially leading to better outcomes of disclosure.

Given the formal nature of therapeutic relationships, it is perhaps unsurprising that the importance of factors when disclosing to health professionals tended to differ the most as compared to the other relationship types. Specifically, factors of most importance to disclosing to health professionals reflected tangible help, including provision of medical care and changes in NSSI (e.g., desire to stop self‐injuring). It may be that individuals expect that as “professionals,” these disclosure recipients should be able to help without stigma or judgment (although this has previously been identified as a barrier to formal disclosures, e.g., Long, 2018), particularly given that the expectation of negative views from others was least important when considering disclosing to health professionals versus other groups. Certainly, previous research has indicated that tangible support can be considered a positive outcome of NSSI disclosure (e.g., Ammerman & McCloskey, 2020; Park et al., 2020).

Interpersonal factors concerned with the reactions of the disclosure recipient, the existing quality of the relationship with them, and the potential impact the disclosure could have on the relationship were perceived to be most important when considering disclosure within personal relationships, as compared to disclosing to health professionals. Indeed, individuals may be less inclined to anticipate relational implications in the latter, perhaps due to having less of a personal and more of a formal connection with professionals. In contrast, it is understandable that potential reactions and impacts on one's relationships were considered more important when considering disclosure to a recipient known personally to an individual. Such disclosures could potentially lead to day‐to‐day disruptions in the person's life and their relationships (Simone & Hamza, 2020).

There were also some differences among the three personal relationship types. For example, though there was a similar pattern of findings for both friends and significant others, factors concerned with help‐seeking and the importance of the recipient knowing about the individual's self‐injury were considered to be more important when disclosing to the latter. Arguably this may be expected if one assumes that a relationship with one's significant other is of a romantic nature and thus more intimate than that of a friend. Therefore, significant other learning about their partner's NSSI could hold deeper implications for their relationship than those of a platonic relationship (e.g., Simone & Hamza, 2020). This finding further highlights that not all NSSI disclosures should be approached in the same way, reflecting recent person‐centered perspectives concerning self‐injury experiences (e.g., Lewis & Hasking 2021).

### 5.1 Implications

The current findings offer insights into how correlates of NSSI disclosure may be prioritized when deciding whether to share one's experience of self‐injury. This potentially indicates that cognitive processes are involved in disclosure decision‐making as suggested by disclosure theorists (Chaurdoir & Fisher, 2010; Greene, 2009). Given that key elements of these theories (e.g., interpersonal factors) appear relevant to the disclosure of NSSI, the application of such models to understanding NSSI disclosure could be further explored in future research.

Additionally, it is possible that disclosing to different people could serve differing functions given the variability in the importance of factors across groups. For example, a health professional may be preferentially sought for tangible aid, while a friend may be disclosed to for social support. As such, aligning the response to an NSSI disclosure given one's relationship with the individual and the reason for them sharing this information is important to meet their needs and to not discourage further disclosure (Simone & Hamza, 2020). That is to say that the findings are indicative of taking a person‐centered approach to NSSI disclosure. In other words, the person sharing their lived experience is placed as the expert of this experience and that when in doubt of the reason for an NSSI disclosure and/or how to respond, recipients should enquire respectfully (Lewis & Hasking, 2021).

Following the above, our findings may provide a first step toward informing the development of resources (e.g., guides, infographics) and other means of support that can be tailored to navigating disclosures to different recipients. In particular, the findings highlight the pragmatic role clinicians could play in supporting clients with lived experience of self‐injury. For example, the way that clinicians should respond to NSSI disclosures (i.e., nonjudgmental, providing requested tangible aid), in addition to collaboratively mentoring their client in navigating disclosures to others, is in line with person‐centered practice recommendations (e.g., Lewis & Hasking, 2019).

### 5.2 Limitations and future research

There are some limitations to bear in mind when considering the findings of this research. For instance, this study used exclusively self‐report cross‐sectional ratings from a nonclinical sample in which participants were asked to rate the importance of the factors in a hypothetical decision to disclose to different parties in a face‐to‐face setting. It is plausible that the relative importance of factors may differ between actual face‐to‐face and other disclosure contexts, such as disclosing to someone online (Frost et al., 2016). Disclosure considerations could also differ across the demographics of those disclosing; the current sample was largely homogenous (e.g., predominantly female) and as such could not capture other potential factors that may be important to more diverse samples. Future research is needed to extend current understandings to different samples and explore the extent to which, and how decisions to disclose NSSI may vary (e.g., across cultures, age groups, in clinical samples). While this study provides preliminary considerations for disclosing NSSI to different people, future research could explore motivations for disclosure across contexts (e.g., in emergency rooms). Similarly, as even infrequent NSSI has been associated with increased risk for adverse outcomes such as suicidality (Whitlock et al., 2013), future research should explore how people's NSSI history (e.g., frequency, medical severity) affects disclosure decisions. Furthermore, we present the average relative importance of factors, which may not reflect how and what considerations might be prioritized in the decision to disclose for a particular individual, in a given situation. Finally, we recognize that factors other than those we examined may play a role in the decision to disclose NSSI; hence, there may be merit in asking people with lived experience of NSSI what they view as important to disclosure via more open‐ended (e.g., interview) approaches (Lewis & Hasking, 2021).

## 6 CONCLUSION

The present study offers initial insight into the importance of a range of factors to the decision of whether to disclose NSSI in informal (e.g., friends) and formal (e.g., health professionals) settings. Notably, there may be unique considerations in particular disclosure contexts. In this way, the present findings set the stage for several theoretical and empirical implications for how NSSI disclosure manifests, which, in turn, can inform efforts to work toward appropriate and effective responding to individuals with lived experience.

## CONFLICT OF INTEREST STATEMENT

The authors declare no conflict of interest.

### 1 PEER REVIEW

The peer review history for this article is available at https://www.webofscience.com/api/gateway/wos/peer-review/10.1002/jclp.23503.

## Data Availability

The data that support the findings of this study are available on request from the corresponding author. The data are not publicly available due to privacy or ethical restrictions.

## ENDNOTE

1 Note that the sum of these percentages exceeds 100 as people could report multiple disclosures.
